# Dosimetric feasibility of hippocampal avoidance whole brain radiotherapy with an MRI‐guided linear accelerator

**DOI:** 10.1002/acm2.13587

**Published:** 2022-03-28

**Authors:** Jasmine A. Graham, Gage Redler, Kirby B. Delozier, Hsiang‐Hsuan Michael Yu, Daniel E. Oliver, Stephen A. Rosenberg

**Affiliations:** ^1^ H. Lee Moffitt Cancer Center and Research Institute Tampa Florida United States

**Keywords:** Dosimetry, HA‐WBRT, MRI linac, Treatment planning

## Abstract

**Purpose/Objective(s):**

Whole brain radiotherapy with hippocampal avoidance (HA‐WBRT) is a technique utilized to treat metastatic brain disease while preserving memory and neurocognitive function. We hypothesized that the treatment planning and delivery of HA‐WBRT plans is feasible with an MRI‐guided linear accelerator (linac) and compared plan results with clinical non‐MRI‐guided C‐Arm linac plans.

**Materials/Methods:**

Twelve HA‐WBRT patients treated on a non‐MRI‐guided C‐Arm linac were selected for retrospective analysis. Treatment plans were developed using a 0.35T MRI‐guided linac system for comparison to clinical plans. Treatment planning goals were defined as provided in the Phase II Trial NRG CC001. MRI‐guided radiotherapy (MRgRT) treatment plans were developed by a dosimetrist and compared with clinical plans. quality assurance (QA) plans were generated and delivered on the MRI‐guided linac to a cylindrical diode detector array. Planning target volume (PTV) coverage was normalized to ∼95% to provide a control point for comparison of dose to the organs at risk.

**Results:**

MRgRT plans were deliverable and met all clinical goals. Mean values demonstrated that the clinical plans were less heterogeneous than MRgRT plans with mean PTV V37.5 Gy of 0.00% and 0.03% (*p* = 0.013), respectively. Average hippocampi maximum doses were 14.19 ± 1.29 Gy and 15.00 ± 1.51 Gy, respectively. The gamma analysis comparing planned and measured doses resulted in a mean of 99.9% ± 0.12% of passing points (3%/2mm criteria). MRgRT plans had an average of 38.33 beams with average total delivery time and beam‐on time of 13.7 (11.2–17.5) min and 4.1 (3.2–5.4) min, respectively. Clinical plan delivery times ranged from 3 to 7 min depending on the number of noncoplanar arcs. Planning time between the clinical and MRgRT plans was comparable.

**Conclusion:**

This study demonstrates that HA‐WBRT can be treated using an MRI‐guided linear accelerator with comparable treatment plan quality and delivery accuracy.

## 1 INTRODUCTION

Whole brain radiotherapy (WBRT) has long been the standard of care treatment for patients with numerous brain metastases^1^ and for prophylactic irradiation of patients with small cell lung carcinoma.^2^, ^3^ The diagnosis of brain metastases historically was an indicator of end‐stage disease and that a patient's treatment would shift to palliative care.^1^ Due to advances in systemic therapy, the brain metastases treatment focus has shifted from increasing survival following brain metastases treatment to maintaining and improving quality of life following diagnosis.^1^ Traditional WBRT has been associated with memory loss and cognitive decline due to hippocampal injury and hypothesized secondary to loss of stem cells in the hippocampus.^4^, ^5^, ^6^ Hippocampal avoidance WBRT (HA‐WBRT) is a new approach to improve quality of life following WBRT.

Based on a multi‐institutional study, RTOG 0933, HA‐WBRT uses volumetrically modulated arc therapy (VMAT) to limit dose to the hippocampi and often utilizes noncoplanar beam angles. This technique has proven to reduce memory loss in patients with brain metastases as assessed most commonly via the Hopkins verbal learning test and other tests of recall.^7^ Studies have shown that conformally avoiding the hippocampus during WBRT preserves memory and quality of life,^8^ and HA‐WBRT has even proven feasible in patients with hippocampal involvement where the tumor is covered and hippocampal dose is minimized.^9^

The dosimetric approaches to HA‐WBRT have been assessed for a variety of treatment modalities. One of the first dosimetric studies was a how‐to guide on HA‐WBRT planning utilizing helical TomoTherapy and linear accelerator (linac) based‐intensity modulated radiation therapy (IMRT).^10^ More recent studies have tested the dosimetric performance using multi‐criteria optimization for VMAT and IMRT to improve the dosimetric quality of these plans.^11^ Wang et al. reported dosimetric efficiency with acceptable plans utilizing Pinnacle's Auto‐Planning module to decrease planning time.^12^ Planning comparisons of 3D‐conformal radiation therapy (CRT), IMRT, and RapidArc (VMAT) were assessed by Wang et al.^13^ They found that the hippocampus was protected best by the 3D‐CRT treatment plan but that the target coverage was lowest. Rong et al. evaluated the dosimetry of IMRT, VMAT, and Helical Tomotherapy for HA‐WBRT and determined that TomoTherapy was the preferred modality for HA‐WBRT due to superior dose distribution (superior homogeneity index).^2^ However TomoTherapy treatments were longer than RapidArc (VMAT) treatments. Proton therapy has also been studied for HA‐WBRT treatments. Stoker et al assessed intensity modulated proton therapy (IMPT) for HA‐WBRT and found that HA IMPT dosimetry was equal to or superior to modulated X‐ray treatments.^14^ IMPT reduced the homogeneity index by approximately 50% compared with X‐ray HA‐WBRT.

The MRI‐linac provides the unique advantage of acquiring daily volumetric MRI images before daily treatments; allowing for physician visualization of the daily anatomy and potential adaption of the treatment based on these images to treat the anatomy of the day more appropriately. For HA‐WBRT, daily adaptation would not likely be necessary but would provide potential advantage allowing for the tracking of brain metastases during treatment. The availability of on‐board daily MR‐guidance may allow us to better ascertain if lesions are responding appropriately during radiotherapy. If not, we could incorporate boosts to lesions with the hope of improving response and durability. However, before pursuing HA‐WBRT, we need to ensure there is dosimetric equipoise between MRI‐guided radiotherapy (MRgRT) and traditional linac‐based treatment. To our knowledge, this is the first dosimetric assessment of HA‐WBRT utilizing an MR‐linac.

## 2 MATERIALS/METHODS

Twelve patients originally treated with HA‐WBRT VMAT plans on a C‐Arm linear accelerator were selected for retrospective planning on a ViewRay MRIdian 0.35T MRI‐guided linac (ViewRay Inc., Cleveland, OH) to investigate feasibility and evaluate dosimetric quality relative to clinical plans. All patients had a T1‐weighted MRI with isotropic 1 mm resolution acquired with gadolinium‐based intravenous contrast and was fused to a planning CT dataset. The physician contoured the left and right hippocampi on the T1 MRI and expanded 5 mm isotropically to provide the hippocampal avoidance structure. The planning target volume (PTV) was expanded 3 mm from the clinical target volume (CTV) excluding the hippocampal avoidance structure. All plans were developed to follow the RTOG 0933 dosimetric criteria:

1. PTV V30 ≥ 95%
2. PTV D2% ≤ 37.5Gy
3. PTV D98% ≥ 25Gy
4. Hippocampi D_max_ ≤ 16Gy
5. Hippocampi D100% ≤ 9Gy
6. Optic Structures D_max_ ≤ 33Gy
7. Lenses D_max_ ≤ 7Gy [RTOG 0933 requests Lens D_max_ <5Gy]

ViewRay treatment planning system (TPS) version 5.4.0.97 was used for treatment planning using a step‐and‐shoot IMRT approach for a MRIdian Viewray 0.35T MRI‐guided linac. MRI‐guided treatment (MRgRT) plans were developed by a dosimetrist with ViewRay TPS experience (plans were further edited by a physicist) and compared with the clinical VMAT plans. This system utilizes a Monte Carlo dose calculation engine that includes magnetic field effects. An isotropic 2 mm dose grid was used to calculate dose with 0.2% uncertainty. Clinical simulation CT scans were used to provide physical density maps for dose calculation. The ViewRay couch model was inserted in each plan to approximate hypothetical treatment positioning.

Clinical DICOM structures were imported for planning in ViewRay. These included targets (CTV and PTV) and organs at risk (OARs) (left/right hippocampi, left/right optic nerve, optic chiasm, left/right lens, left/right orbit). Additional planning structures were generated within the ViewRay TPS: skin (to define nonsupport structure regions for dose calculation consideration), normal tissue (skin minus PTV), a ring structure to control dose spillage beyond PTV (region between PTV + 3 mm and PTV + 8 mm), as well as hippocampal avoidance region (hippocampi + 5 mm). Occasionally, tuning structures were added during the optimization process to control unwanted hot/cold spots. The isocenter for all coplanar IMRT fields was manually placed near the center of the ventricles.

The optimization approach and cost function parameters were tailored to each patient. However, the general approach for MRgRT plans is summarized in Table 1 and was as follows. One goal to increase dose to PTV (i.e., increase 30Gy target coverage) and one goal to decrease dose to PTV (i.e., decrease 33Gy heterogeneity), each with nearly equal importance and power (average/range for importance and power was 66/10–250 and 1.6/1.5–2.0, respectively). Goals were added to decrease dose to optic nerves/chiasm (with an average/range for dose limit, importance, and power of 31.7/28–33 Gy, 6.5/1–40, and 1/1–1.3, respectively). Two goals to reduce dose in the hippocampi were used to limit both D_max_ and D_100%_. The dose level average/range used for these goals was 15.4/13.0–16.0 Gy and 8.5/7.9–9.0 Gy, respectively (importance of 28.3/1–100 and 13.9/1–100, respectively; power of 1 for all). Lens dose was given lower priority, but all plans had low importance/power (∼1/∼1) goals to decrease dose (average/range of dose level used was 4.6/4.3–5.0 Gy) to reduce unnecessary dose to lenses without compromising other aspects of plan quality. Similar low importance/power goals were added to decrease dose above ∼28–29 Gy to normal tissue and/or PTV ring structures to enhance conformality and reduce dose to the scalp. All plans were normalized to ensure PTV V30Gy = 95% (±0.5%) to facilitate direct comparison of all other dosimetric goals.

**TABLE 1:** Optimization approach for MRI‐guided radiotherapy (MRgRT) plans

Structure	Purpose	Type	Importance	Power	Threshold
PTV	Increase 30 Gy target coverage	Increase dose	66 (10–250)	1.6 (1.5–2.0)	30.1 Gy (30–31 Gy)
PTV	Decrease 33 Gy heterogeneity	Decrease dose	66 (10–250)	1.6 (1.5–2.0)	33.1 Gy (33–34 Gy)
Optic nerves/chiasm	Limit D_max_	Decrease dose	6.5 (1–4)	1 (1–1.3)	31.7 Gy (28–33 Gy)
Hippocampi	Limit D_max_	Decrease dose	28.3 (1–100)	1	15.4 Gy (13–16 Gy)
Hippocampi	Limit D_100%_	Decrease dose	13.9 (1–100)	1	8.5Gy (7.9–9.0 Gy)
Lenses	Approach as low as reasonably achievable (ALARA) dose	Decrease dose	1.3 (1–3)	1	4.6 Gy (4.3–5.0 Gy)
Normal tissue/PTV ring structures	Provide conformality and reduce scalp dose	Decrease dose	1	1	29 Gy

Abbreviation: PTV, planning target volume.

A large number of fixed‐fields (average of 38.7 beams; range of 36–42 beams) were used to closely approximate treatment with an arc approach. Left‐posterior and right‐posterior oblique angles (∼90–135° and ∼225–270°, respectively) were not used in order to avoid beams treating through longer diagonals along the couch as the ViewRay table‐top is relatively high density. In order to allow for maximum field modulation, the IMRT efficiency parameter was kept lower (close to 1), and the number of segments per plan was allowed to be relatively high (average of 130.7 segments; range of 100–171 segments). Bixel size was set to 0.3 cm with 50 000 histories per cm^2^.

**FIGURE 1:**
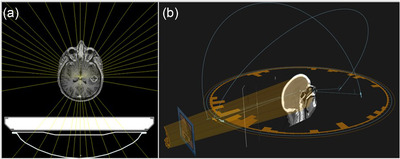
Representative beam arrangements for (a) MRI‐guided radiotherapy (MRgRT) co‐planar fixed‐field IMRT and (b) noncoplanar volumetrically modulated arc therapy (VMAT) on a conventional c‐arm linear accelerator

Pinnacle^3^ version 14.0 (Philips, Fitchburg, WI) was used for developing clinical VMAT plans. Plans were generated for delivery on a c‐arm linear accelerator (Varian Medical Systems, Inc., Palo Alto, CA) with the collapsed‐cone convolution superposition dose engine with a 3 mm isotropic dose grid. The original patient treatment plans were normalized to 95% coverage for one‐to‐one comparisons. Typical VMAT beam arrangement consisted of two noncoplanar (couch angles of 300 and 60^o^) and two coplanar angles (Figure 1b). For two cases, noncoplanar beam angles were not utilized, but rather four coplanar VMAT arcs were used. All plans were normalized to ensure PTV V30Gy = 95% (±0.5%) to facilitate direct comparison of all other dosimetric goals.

QA plans for a subset of the MRgRT plans were generated by recalculating treatment beams on a cylindrical phantom representing the MR‐compatible ArcCHECK‐magnetic resonance (MR) (Sun Nuclear Coproration, Melbourne, FL). These plans were then delivered with the MRI‐guided linac and measured with the helical diode detector array of the ArcCHECK‐MR to verify deliverability of planned dose.

Dose volume histograms metrics were extracted from the MRgRT and clinical VMAT treatment plans for the PTV, CTV, optic structures, hippocampi, and lenses. A paired two‐tailed t‐test was calculated from the metrics to provide statistical significance where *p*‐values ≤ 0.05 were considered statistically significant.

## 3 RESULTS

**FIGURE 2:**
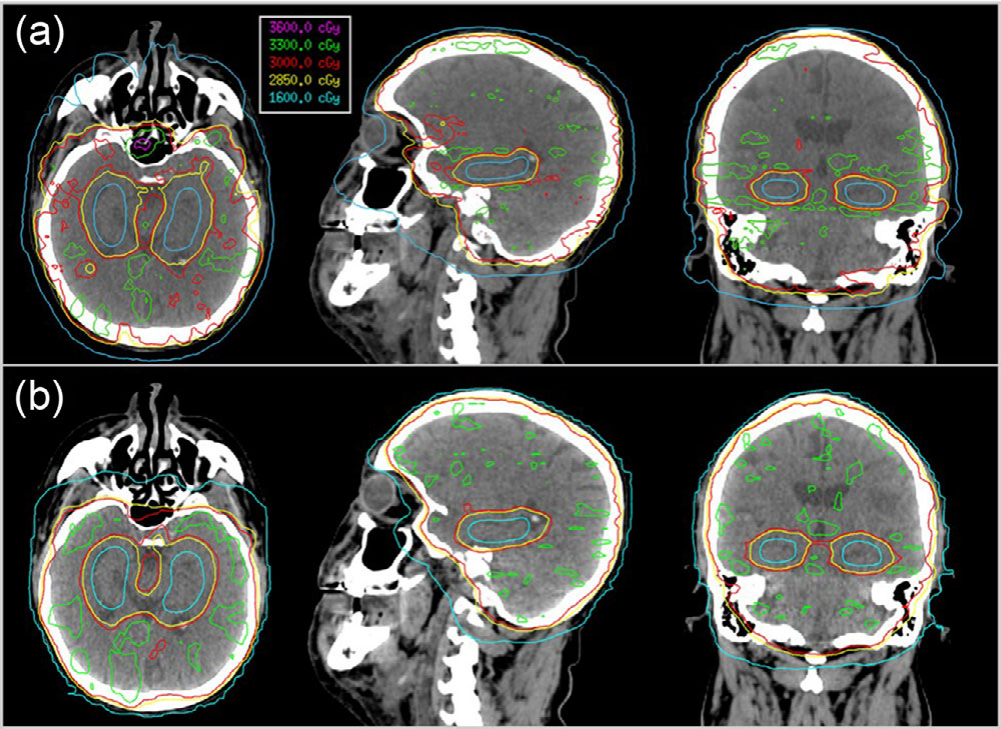
Comparison of achieved dose distributions for hippocampal avoidance (HA) whole brain radiotherapy (WBRT) in a representative patient plan. (a) MRI‐guided radiotherapy (MRgRT) with co‐planar fixed‐field IMRT. (b) Clinical plan with noncoplanar volumetrically modulated arc therapy (VMAT) on a conventional c‐arm linear accelerator

**FIGURE 3:**
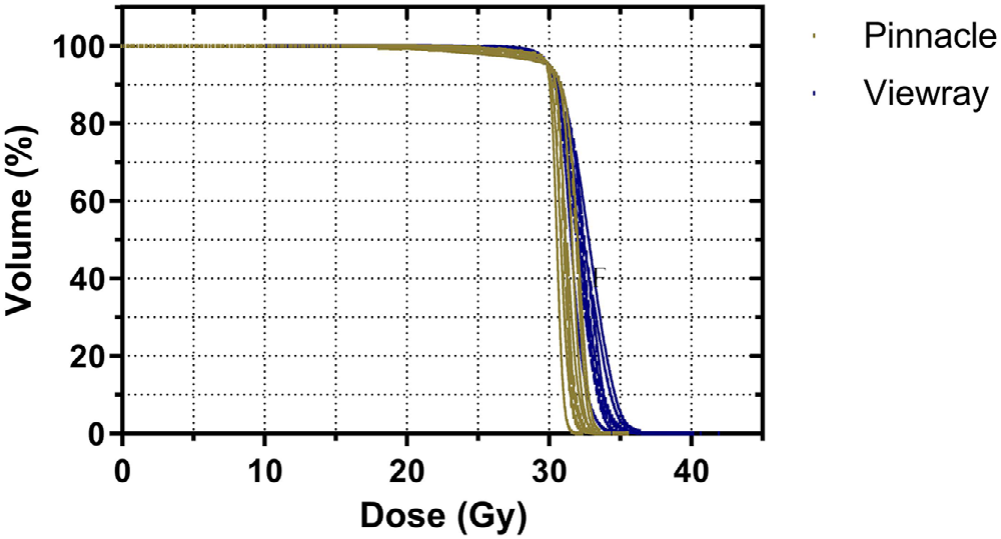
Planning target volume (PTV) dose volume histogram (DVH) comparisons for all cases. MR‐linac plans are in blue and clinical volumetrically modulated arc therapy (VMAT) plans are in gold

The MRgRT plans were all deliverable and met the previously described RTOG 0933 compliance criteria and clinical goals. Figure 2 shows a comparison of the dose distributions for MRgRT (A) and clinical VMAT (B) plans for one patient. The mean values demonstrated that the clinical VMAT plans were more homogeneous than MRgRT plans. The D2% ranged from 33.70 Gy to 35.65 Gy for MRgRT plans compared with 32.73 Gy to 35.01 Gy for clinical VMAT plans. This trend was statistically significant (*p* = 0.044). Figure 3 demonstrates that MRgRT plans exhibited a slower falloff with dose resulting in higher dose to the PTV.

RTOG 0933 hippocampal sparing requirements were achieved with the MRgRT plans. The average hippocampi maximum doses were 14.19 ± 1.29 Gy and 15.00 ± 1.51 Gy (*p* = 0.213) for clinical VMAT versus MRgRT plans, respectively. The average hippocampi D100% were 8.62 ± 0.51 Gy and 7.92 ± 0.33 Gy, respectively. This was statistically significant (*p* = 0.0026). The average maximum doses to 0.03 cc of the optic structures were 30.94 ± 1.10 Gy and 31.26 ± 1.97 Gy (p = 0.411), respectively (see Table 2).

**TABLE 2:** Average dosimetric values for clinical VMAT plans and MRgRT (static field IMRT) plans

	Average values		
	Clinical VMAT	MRgRT	*p‐*Value
**PTV V30**	95.02% ± 0.00%	95.00% ± 0.03%	*0.243*
**PTV V37.5**	0.00% ± 0.00%	0.03% ± 0.03%	*0.013*
**PTV V25**	99.50% ± 0.66%	99.59% ± 0.16%	*0.589*
**CTV V28.5**	96.26% ± 1.52%	96.08% ± 0.41%	*0.724*
**Hippocampi D_max_ (0.03 cc)**	14.19 Gy ± 1.29 Gy	15.00 Gy ± 1.51 Gy	*0.213*
**Hippocampi D_100%_**	8.62 Gy ± 0.51 Gy	7.92 Gy ± 0.33 Gy	*0.003*
**Optic structures D_max_ (0.03 cc)**	30.94 Gy ± 1.10 Gy	31.26 Gy ± 1.97 Gy	*0.411*
**Lens D_max_ (0.03 cc)**	5.64 Gy ± 2.53 Gy	6.82 Gy ± 4.21 Gy	*0.086*

*Note*: PTV V37.5 and Hippocampi D_100%_ are statistically significant.

Abbreviations: CTV, clinical target volume; MRgRT, MRI‐guided radiotherapy; PTV, planning target volume; VMAT, volumetrically modulated arc therapy.

Quality assurance was performed for a subset of plans and the gamma analysis comparing planned and measured doses resulted in a mean of 99.9 ± 0.12% of passing points (3%/2mm criteria) for all plans. The MRgRT plans had an average of 38.33 beams with total delivery time ranging from 11.2 to 17.5 min and 4.13 min (average) for total beam on time. Whereas clinical plans had average delivery times of 3–7 min depending on the number of coplanar arcs. The planning time between the clinical and MRgRT plans was comparable.

## 4 DISCUSSION

Although the MRgRT plan doses were less homogeneous than the clinical VMAT plans (average PTV D2% = 34.79 vs. 34.19 Gy [*p* = 0.044]), the MRgRT plans were deliverable and provided equivalent PTV coverage compared to the clinical VMAT plans (as all plans were normalized to have approximately equivalent coverage of V100 ∼95%). Additionally, RTOG 0933 compliance criteria were successfully met. The benefit of the traditional linac plans is that the plans would be delivered more quickly.

There have been other dosimetric studies on planning approaches for HA‐WBRT. Initially, dosimetric studies focused on helical TomoTherapy and linac‐based IMRT. Gondi et al. published a how‐to guide on HA‐WBRT planning approaches comparing helical TomoTherapy and LINAC‐based IMRT.^10^ This study demonstrated a mean hippocampal volume of 3.3 cc (2.1% of the whole brain PTV). TomoTherapy hippocampus doses were lower than linac‐based: 5.5 Gy and 12.8 Gy (median and maximum) versus 7.8 Gy and 15.3 Gy (median and maximum), respectively. For comparison, our mean hippocampal volumes were 3.59 cc. The superiority of the TomoTherapy plans specific to the hippocampal avoidance was attributed to the faster dose fall‐off that this TomoTherapy facilitates. Although the MRgRT does not offer helical delivery, it does offer a double‐focused multileaf collimator (MLC), which may reduce penumbra and low dose outside the target due to MLC leakage.

Another study by Rong et al. compared linac‐based IMRT, VMAT, and Helical TomoTherapy dosimetry for HA‐WBRT plans. In this study, the TomoTherapy plans provided a significantly superior homogeneity index compared with linac‐based IMRT (most inferior homogeneity index) and VMAT.

Many comparison studies of VMAT versus IMRT techniques for other sites have been evaluated. Generally, VMAT plans require extended planning time (likely due to the speed of the optimization algorithm), a lower number of monitor unit (MU), and a faster treatment time.^15^, ^16^ VMAT plans have also been associated with better homogeneity and conformity index for cervix/uterus cases.^17^ One disadvantage of these plans in our study was that the MRgRT IMRT plans resulted in a longer beam‐on time.

To our knowledge, this is the first study assessing HA‐WBRT planning and delivery with an MRI‐linac. The potential advantages are not necessarily related to delivery or planning but the imaging capabilities of the MR‐guided delivery. These imaging advantages are currently being expanded and optimized as new technologies and capabilities are introduced into the MRI‐linac workflow. The MRI‐linac now offers the capability of administering contrast during a treatment on the MRI‐linac. The introduction of contrast for brain treatment could facilitate the tracking of brain metastases during treatment. Utilizing the MRIdian including T1+ contrast and DWI (experimental) in low field could provide daily MR‐guided images which will facilitate tracking lesion response during treatment. Lesions that do not respond favorably during treatment will be assessed for a stereotactic boost. There are, of course, additional technological issues that must be overcome including resolution and sequence inadequacies that could be alleviated via head coils and robust head immobilization techniques. However, this region is currently being explored with glioblastoma (GBM) with great interest.^18^

A different approach is HA‐WBRT with simultaneous integrated boost. Lebow et al. published their experience treating with HA‐WBRT combined with simultaneous integrated boost, which demonstrated a combination of local control, higher dose to disease and sterilization of microscopic disease while keeping hippocampal doses and acute toxicities low.^5^

Regardless, before any clinical studies were pursued, we needed to ensure that MRgRT provided equivalent dosimetric benefit to the patient. Our analysis shows that MRgRT may be used to deliver HA‐WBRT safely and effectively and opens an avenue for exploration of biological response during radiotherapy for brain metastasis.

## 5 CONCLUSION

This study demonstrates that HA‐WBRT can be treated using an MRI‐guided linear accelerator with comparable treatment plan quality and delivery accuracy. Given the equitable dosimetric outcomes to traditional CT‐based plans, the use of MRgRT for hippocampal sparing WBRT opens the possibility of radiomic analysis and potential adaptive treatments (i.e., boost) to lesions based on disease response.

## CONFLICT OF INTEREST

Dr. Rosenberg has performed consulting for Novocure. He also has research funding from ViewRay and serves on the Lung Research Consortium Advisory Board (noncompensated).

## FUNDING INFORMATION

ViewRay

## AUTHOR CONTRIBUTIONS

Jasmine A. Graham (previously Jasmine A. Oliver) was instrumental in research development and design. Gage Redler edited manuscript, supplied methods information, was instrumental in research development and design, and provided QA results. Kirby B. Delozier developed treatment plans for analysis. Hsiang‐Hsuan Michael Yu and Daniel E. Oliver reviewed/edited manuscript and provided feedback. Stephen A. Rosenberg reviewed/edited manuscript, provided feedback, and was instrumental in research development and design.

## Data Availability

Data are available upon request.
